# Fluoxetine-induced perinatal morbidity in a sheep model

**DOI:** 10.3389/fmed.2022.955560

**Published:** 2022-08-04

**Authors:** Rafael R. Domingues, Adam D. Beard, Meghan K. Connelly, Milo C. Wiltbank, Laura L. Hernandez

**Affiliations:** ^1^Department of Animal and Dairy Sciences, University of Wisconsin-Madison, Madison, WI, United States; ^2^Endocrinology and Reproductive Physiology Program, University of Wisconsin-Madison, Madison, WI, United States

**Keywords:** intrauterine growth restriction, neonatal morbidity, preterm labor, acid-base balance, psychotropic drugs, selective serotonin reuptake inhibitor

## Abstract

Selective serotonin reuptake inhibitors (SSRI) are the most common antidepressants used by pregnant women. However, adverse pregnancy outcomes have been described in women taking SSRI during pregnancy—placental lesions, premature birth, poor neonatal adaptation. We aimed to investigate the effects of fluoxetine (Prozac^®^ most commonly used SSRI) treatment during the last month of gestation on pregnancy complications, placental and neonatal health in a non-depressed sheep model. On day 119 ± 1 postbreeding (experimental day 0; E0) of a 151-day expected gestation, Hampshire ewes were randomly assigned to receive fluoxetine (*n* = 9 ewes, 15 lambs; daily intravenously treatment with 10 mg/kg on E0 and E1 and 5 mg/kg daily thereafter until parturition) or to a control group (*n* = 10; 14 lambs; vehicle only). Blood samples from ewes were collected throughout the experimental period and postpartum; blood from lambs were collected postpartum. Analysis of variance was used for statistical analysis. Fluoxetine treatment reduced placentome growth during the last month of pregnancy. Gestation length was decreased by 4.5 days in fluoxetine-treated ewes. Birthweight was reduced in lambs exposed to fluoxetine *in utero*; weights remained decreased until postnatal day 3. Placentome diameter by birthweight ratio was not different between groups suggesting that the decreased placentome diameter was accompanied by decreased lamb birthweight. During the first week postnatal, lambs exposed to fluoxetine *in utero* had decreased blood pH and decreased total carbon dioxide, bicarbonate, and base excess and increased lactate (days 3–6), collectively indicative of metabolic acidemia. Additionally, ionized calcium was decreased between postnatal days 0 to 4 in lambs exposed to fluoxetine *in utero*. Using a non-depressed animal model clearly defines a role for SSRI on the occurrence of perinatal complications and neonatal morbidity. The decreased placentome diameter, shortened gestation, decreased birthweight, decreased calcium levels, and neonatal acidemia suggest the occurrence of intrauterine growth restriction. The persistence of neonatal acidemia for several days postpartum suggests poor neonatal adaptation to extrauterine environment.

## Introduction

Selective serotonin reuptake inhibitors (SSRI) are the primary class of antidepressants prescribed to treat depression in pregnant women and fluoxetine (Prozac^®^), the first SSRI introduced to the market, is still one of the most popular antidepressants worldwide (1). Approximately 15% of pregnant women suffer from depression and 8–13% are prescribed antidepressants during gestation (2, 3). Although SSRI use during pregnancy has increased in the past 20 years, numerous studies have described an increase in adverse outcomes for both mother and infant related to SSRI use (4–6). However, interpretation of these adverse pregnancy outcomes in women is confounded by the effects of underlying depression itself (5).

Circulating serotonin is primarily transported by platelets upon its uptake by serotonin transporter (SERT). Inhibition of SERT by SSRI leads to decreased platelet concentrations of serotonin (as measured in serum or whole blood samples) while plasma concentrations are increased (7, 8). Elevated plasma concentrations of serotonin during SSRI treatment potentially alter placental function. The involvement of serotonin in intrauterine growth restriction (IUGR) has been investigated (9–11). Serotonin is a potent vasoconstrictor; therefore increased serotonergic signaling decreases uterine/placental vascular perfusion (12, 13) giving rise to placental complications such as placental insufficiency, the main cause of IUGR (10, 14). Indeed, placentae from women undergoing SSRI treatment during pregnancy have several lesions of malperfusion in the maternal and fetal sides of the placenta along with decreased birthweight (15). However, the effects of SSRI on placenta size/growth, another landmark of IUGR (16, 17), are still poorly understood.

Neonatal disorders associated with intrauterine exposure to SSRI are numerous and associated with increase neonatal morbidity (4, 5, 18). Besides fluoxetine's effects on neurodevelopment (19, 20), treatment during pregnancy is related to increased neonatal risk for adverse respiratory and cardiovascular functions (21, 22). The potential decreased uterine/placental vascular perfusion associated with compromised neonatal respiratory and cardiovascular functions caused by maternal SSRI treatment during pregnancy may result in inadequate oxygen and carbon dioxide exchange in the peripartum period. However, little is known about the effects of intrauterine exposure to SSRI on neonatal blood gas and acid-base homeostasis.

Because of the potential adverse effects of SSRI on placental function and the neonatal cardiorespiratory system, we investigated the effects of fluoxetine exposure during the last month of gestation on placental growth, pregnancy outcomes, and neonatal health in an ovine model. We aimed to (1) explore the role of fluoxetine on the occurrence of IUGR, decreased gestation length, decreased birthweight, neonatal morbidity and (2) evaluate the effect of fluoxetine on placental growth in a non-depressed animal model. We hypothesized that fluoxetine treatment during late pregnancy (1) decreases gestation length, (2) causes IUGR and (3) affects neonatal health.

## Materials and methods

### Animal management

Timed-bred multiparous Hampshire ewes (4.4 ± 0.4 years old) were obtained from the Arlington Sheep Research Unit from the University of Wisconsin-Madison. Beginning on day 112 ± 1 postbreeding, 20 pregnant ewes were housed in individual pens at the Livestock Laboratory at the University of Wisconsin-Madison and maintained at a constant temperature at 18°C and a 14/10 hour light/dark cycle. All ewes received *ad libitum* access to water and were individually fed haylage, whole-shell corn, and mineral supplement based on live weight according to National Research Council Nutrient Requirements for pregnant ewes (23). After 7 days postpartum, ewes and lambs returned to the Arlington Sheep Research Unit and were grouped housed in an open shelter under natural light.

On day 117 ± 1 postbreeding, jugular catheters were placed in all ewes for intravenous treatment and blood collection. The catheter was aseptically placed, fixated to the neck and protected by a bandage. Catheter patency was maintained during the entire period of treatment/ blood collection.

Ewes were pregnant with a range of one to three fetuses; the average number of fetuses was not different between groups (*p* = 0.5; 1.6 ± 0.2 vs. 1.8 ± 0.2). Delivery was assisted as needed and lambs were fed colostrum by assisted nursing or bottle-fed fresh colostrum from their dam.

### Experimental design

On day 119 ± 1 postbreeding (exp. day 0) of a 151 day expected gestation, ewes were randomly assigned to control or fluoxetine groups. Fluoxetine-treated ewes received fluoxetine hydrochloride (C845, AK Scientific, Union City, California, USA) at 10 mg/kg on exp. days 0 and 1 and at 5 mg/kg daily thereafter until parturition. Fluoxetine dosage was based on a previous experiment from our laboratory aiming to be representative of systemic fluoxetine concentrations in humans. Lyophilized fluoxetine was reconstituted daily in ethanol and diluted into 0.9% NaCl saline (07983-02, Hospira, Lake Forest, Illinois, USA) to the appropriate concentration for each ewe based on body weight. Final ethanol concentration was <3.5%. Body weight was assessed weekly and fluoxetine dose was adjusted accordingly. Ewes in the control group received saline + ethanol at similar ethanol concentration to fluoxetine-treated ewes. All ewes were treated at a continuous infusion rate of 200 mL for 15 min using an automated mini pump (Heska Vet/IV 2.2, Heska, Loveland, Colorado, USA).

### Transabdominal ultrasonography

Placentome (functional unit of placenta in sheep) diameter was evaluated by transabdominal ultrasonography (MindrayZ5, Nanshan, China; 7.5 MHz transducer) (24). A baseline assessment was made prior to treatment (exp. day −1) followed by weekly assessments thereafter (E7, 14, and 21). At least three placentomes were measured per ewe in each evaluation.

### Blood and milk collection

Blood samples from ewes were collected from jugular catheters immediately before each treatment during the prepartum period and for 6 days postpartum. An additional blood sample from ewes was collected within 30 min of parturition. Lambs had a jugular blood sample collected within 25 min of birth (before colostrum intake) and daily for the 6 days postpartum. Blood samples were immediately used for blood gas analysis; remaining blood was centrifuged at 2,000 g for 15 min and serum was stored at −20°C until assayed. Colostrum was collected within 30 min of parturition (before lamb intake) and milk was collected on postpartum day 6.

### Hormone assays and blood gas analysis

Serum serotonin was determined by EIA (IM1749, Beckman Coulter, Czech Republic). The intra- and inter-assay CV were 4.2 and 7.4%, respectively. Serum lactate was determined using Catachem reagents (C454-01, Oxford, Connecticut, USA) on ChemWell-T analyzer. The intra-assay CV was 3.1%. Colostrum and milk calcium concentrations were determined as described (25). The intra-assay CV was 8.0%.

Blood gas analysis was carried out immediately after jugular blood collection using a portable clinical analyzer (i-STAT, Abbott Laboratories, Abbot Park, Illinois, USA) with a CG8+ cartridge. Blood pH, partial pressure of carbon dioxide, bicarbonate, total carbon dioxide, base excess, and ionized calcium were analyzed. Since venous blood was collected, oxygen saturation and oxygen partial pressure data were not used.

### Statistical analysis

All statistical analysis was performed using SAS (version 9.4; SAS Institute Inc., Cary, North Carolina, USA). Data were analyzed with PROC MIXED procedure using one-way ANOVA and two-way ANOVA for repeated measures. Tukey HSH was used for *post hoc* comparisons. Studentized residuals with deviations from assumptions of normality and/or homogeneity of variance were transformed into square root, logarithms, or ranks. Survival analysis was assessed with PROC LIFETEST using Wilcoxon test. A probability of ≤0.05 indicated a difference was significant and a probability between >0.05 and ≤0.1 was considered a tendency for significance. Data are presented as the mean ± standard error of mean (SEM) unless otherwise indicated.

## Results

A fluoxetine-treated ewe with triplets had a foot abscess, stopped eating haylage, and was treated with antibiotics for 5 days during prepartum; therefore, this ewe was not included in the analysis. The same ewe had dystocia; one of the lambs was stillborn and the other two died within 18 h of birth. Anatomopathological findings suggested pneumonia and sepsis. Two lambs (one lamb per ewe) from saline-treated ewes with twin gestation were stillborn due to dystocia. Data from these animals were not used in the final analysis. Overall, 10 control and 9 fluoxetine-treated ewes and 14 and 15 lambs from saline and fluoxetine groups, respectively, were included in the final analysis.

**Figure 1:**
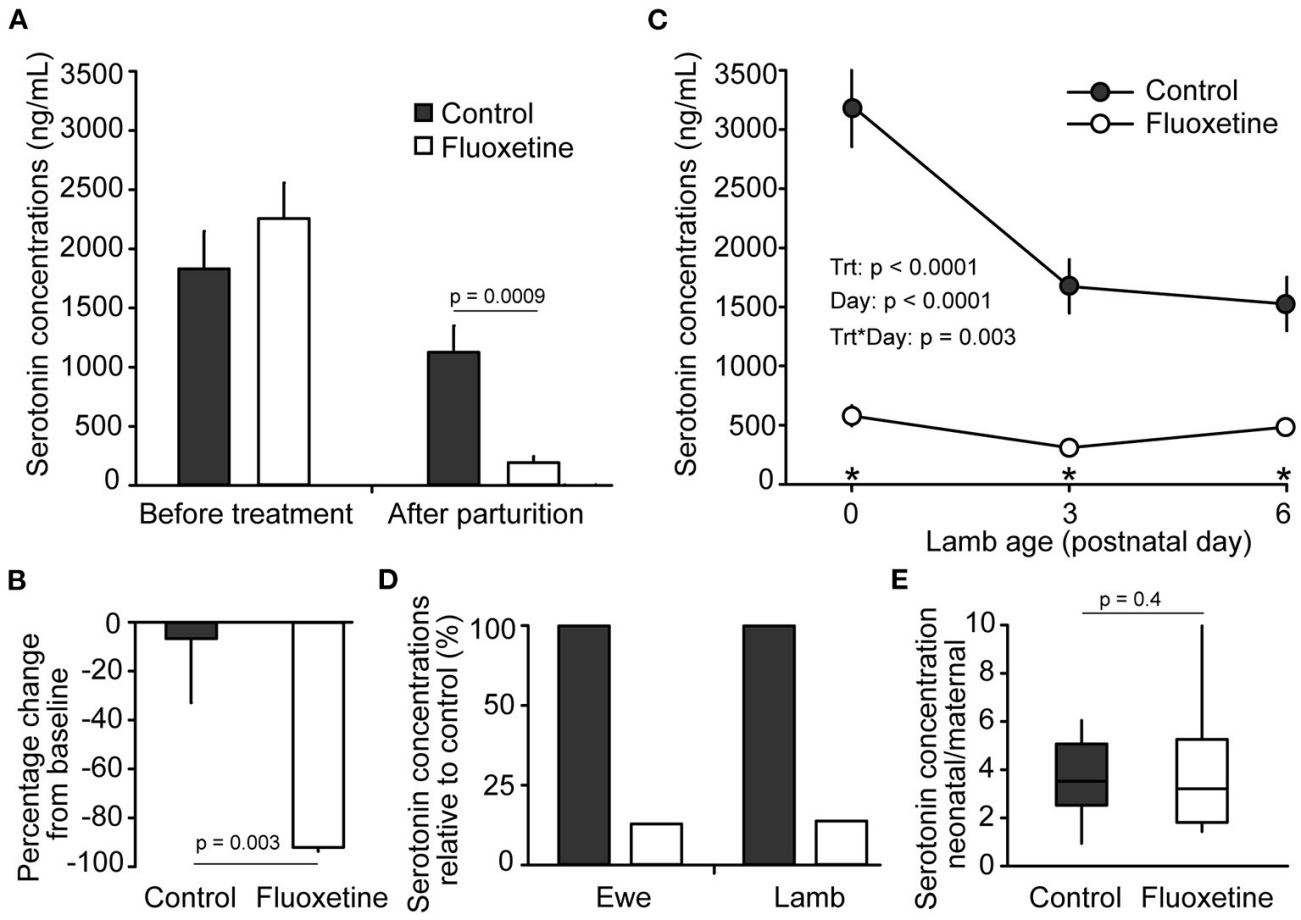
Serotonin concentrations. **(A)** Serum serotonin concentrations in ewes before treatment (baseline) and after parturition. Days from beginning of treatment until parturition varied between 26 to 34 days for the control group and 24 to 31 days for the fluoxetine group. **(B)** Pecentage change in serum serotonin conentrations in ewes between baseline and after parturition. **(C)** Serum serotonin concentrations in lambs. Blood sample on postnatal day 0 was collected before colostrum ingestion. **(D)** Serum serotonin concentrations in ewes and lambs relative to control group on postnatal day 0. **(E)** Neonatal/maternal ratio of serum serotonin concentrations on postnatal day 0; serotonin concentratations in each lamb was divided by serotonin concentrations in their respective dam. *Indicates significant difference between groups.

Fluoxetine treatment decreased serum serotonin concentrations in ewes and lambs (Figure 1). Serotonin concentrations were greater in lambs than in their respective dam on postnatal day 0 (*P* < 0.0007) for both control and fluoxetine groups. However, fluoxetine did not affect the neonatal/maternal ratio of serotonin. Additionally, the decrease in serum serotonin concentrations between control and fluoxetine groups was similar for ewes and lambs (82.9 and 81.6% reduction relative to control, respectively).

**Figure 2:**
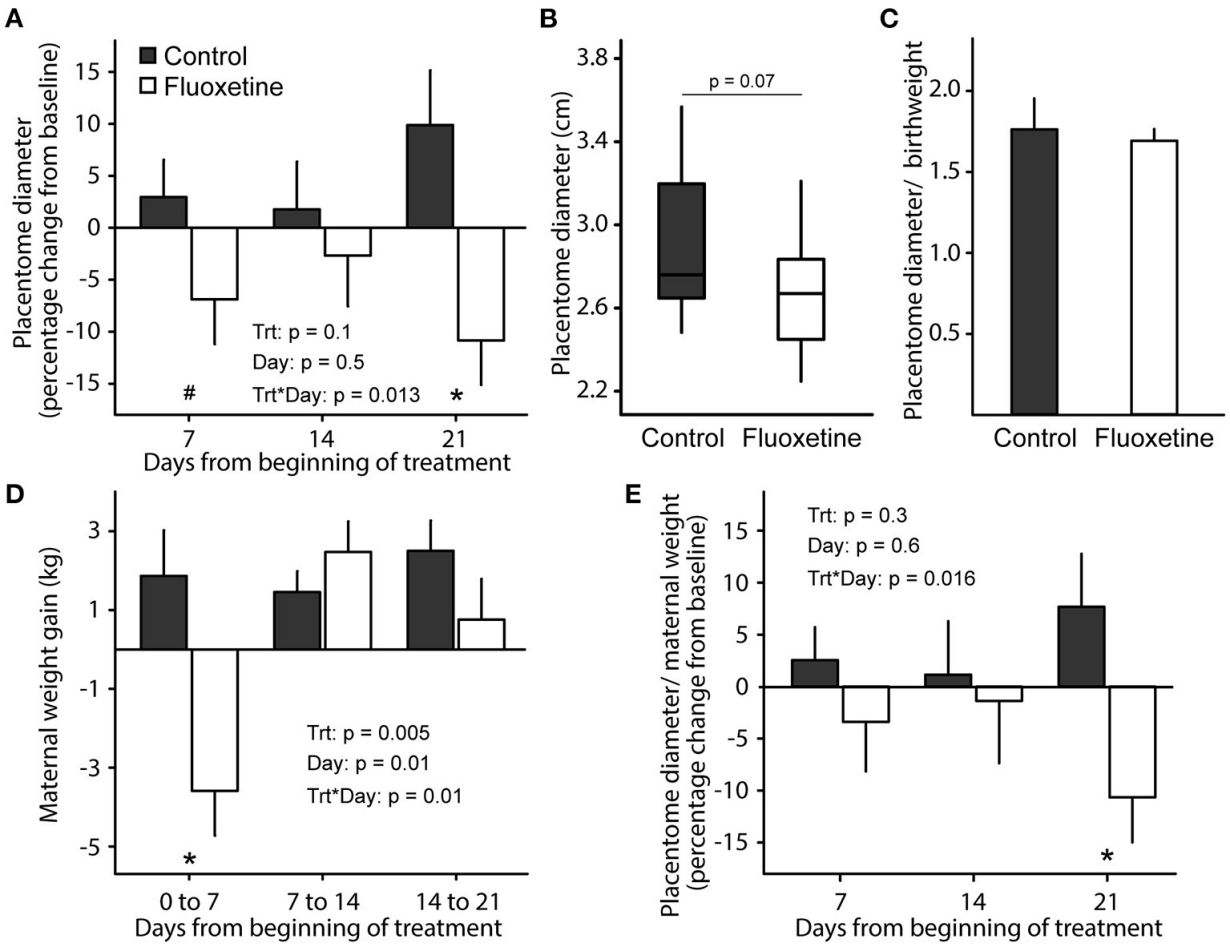
Placentome diameter. **(A)** Placentome diameter throughout experimental period relative to pretreatment. **(B)** Boxplot of placentome diameter on experimental day 21 (gestational day 140 ± 1). Boxplot depicts median, 10th, 25th, 75th, and 90th percentile. **(C)** Placentome diameter by lamb birthweight ratio. **(D)** Maternal weight change from baseline. **(E)** Placentome diameter by maternal weight ratio change from baseline. Baseline maternal weight and placentome diameter were assessed on experimental day −1 (gestational day 118 ± 1). *indicates significant difference between groups and ^#^indicates a tendency for significance.

In the cotyledonary placenta of sheep, maternal-fetal exchange occurs exclusively at the placentome, the functional unit of placenta in ruminants (24). Placentome diameter was similar (*P* > 0.1) between groups before onset of treatment (not shown). After the onset of fluoxetine treatment placentome growth significantly decreased in fluoxetine-treated ewes while it increased in control animals (Figure 2). Mean placentome diameter was 9.5% smaller (*P* = 0.07) in ewes treated with fluoxetine than controls, although there was no difference in placentome diameter (exp. day 21) by lamb birthweight ratio indicating that reduced placentome growth was accompanied by reduced fetal growth.

Fluoxetine-treated ewes lost weight (*P* = 0.0026) compared to controls during the first week of treatment but had similar weight gain as controls from Day 7 until parturition (Figure 2). Similarly, maternal feed intake was decreased (*P* = 0.0005) in the fluoxetine group only during the first week of treatment (4.1 ± 0.04 and 3.6 ± 0.1 kg/day averaged from exp. day 0 to 7 for control and fluoxetine groups, respectively; not shown). In the control group, placentome diameter by maternal weight ratio increased during the 21-day treatment period; conversely, it decreased in fluoxetine-treated ewes indicating reduced placentome growth was occurring even when ewes were gaining weight.

**Figure 3:**
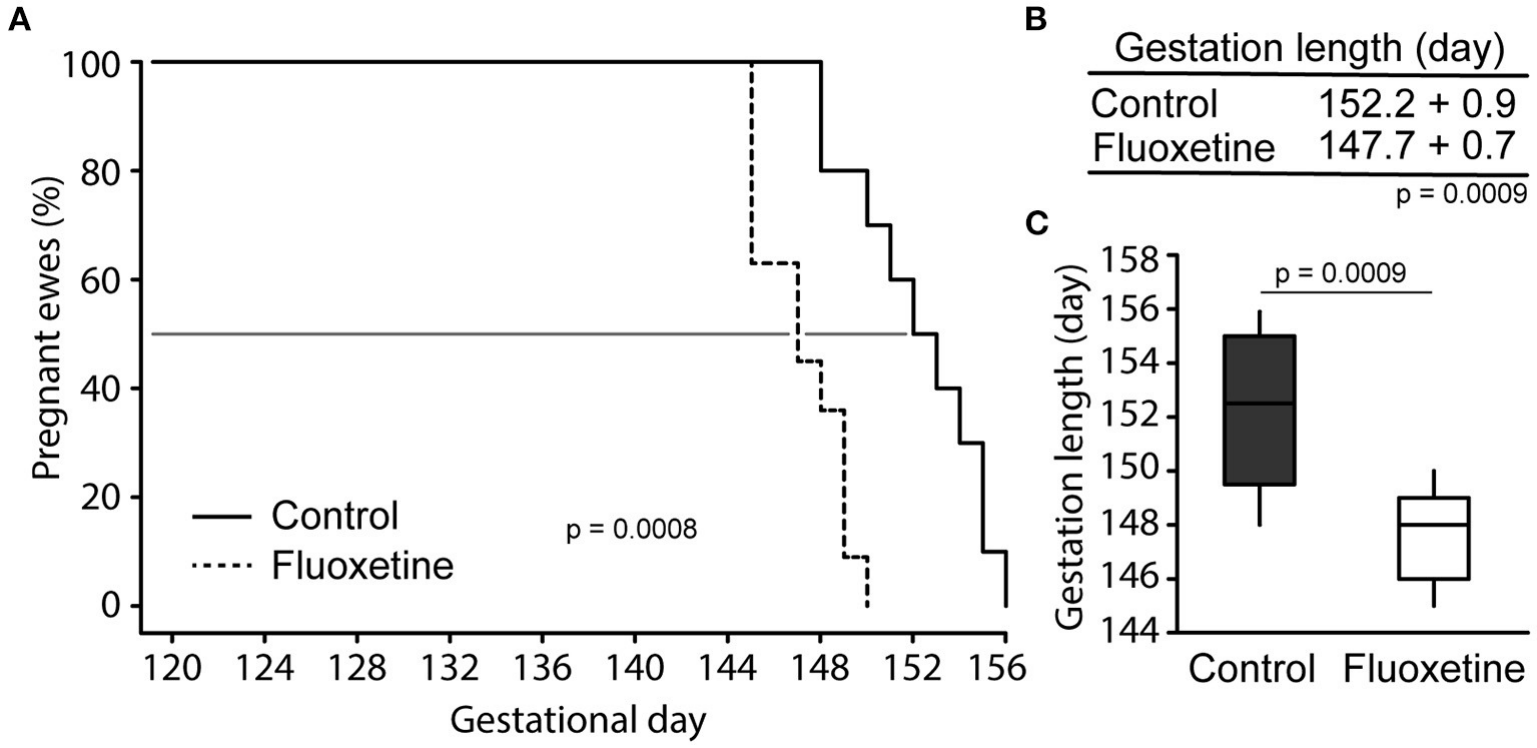
Pregnancy length. **(A)** Survival analysis depicting number of animals giving birth each day. The 50% point is shown. **(B)** Mean gestation length. **(C)** Boxplot of gestation length. Boxplot depicts median, 10th, 25th, 75th, and 90th percentile.

**Figure 4:**
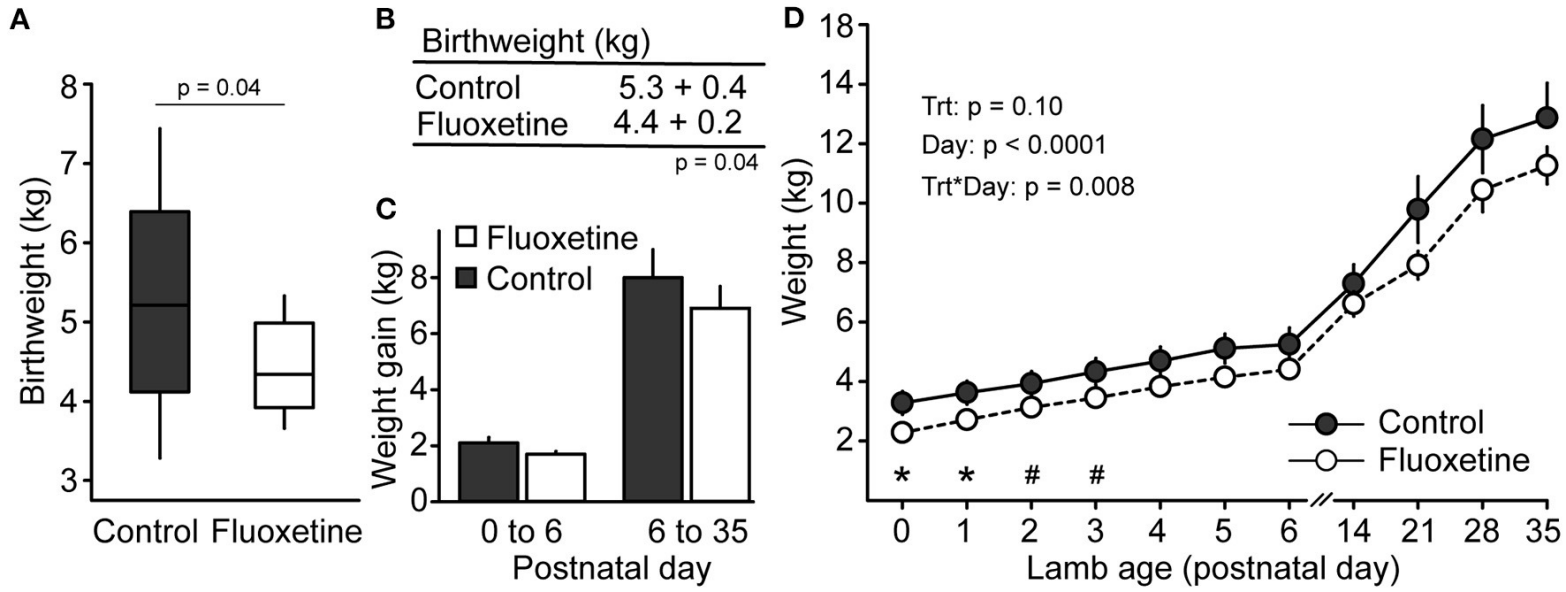
Lamb weight. **(A)** Boxplot of neonatal birthweight. Boxplot depicts median, 10th, 25th, 75th, and 90th percentile. **(B)** Mean lamb birthweight. **(C)** Lamb weight gain. **(D)** Lamb weight during the evaluated period. *Indicates significant difference between groups and ^#^indicates a tendency for significance.

Fluoxetine treatment decreased mean gestation length (Figure 3). Lamb weight was reduced in lambs born to fluoxetine-treated ewes at birth and on postnatal days 1–3 (Figure 4). Lamb weight gain until day 35 was not different between groups.

**Figure 5:**
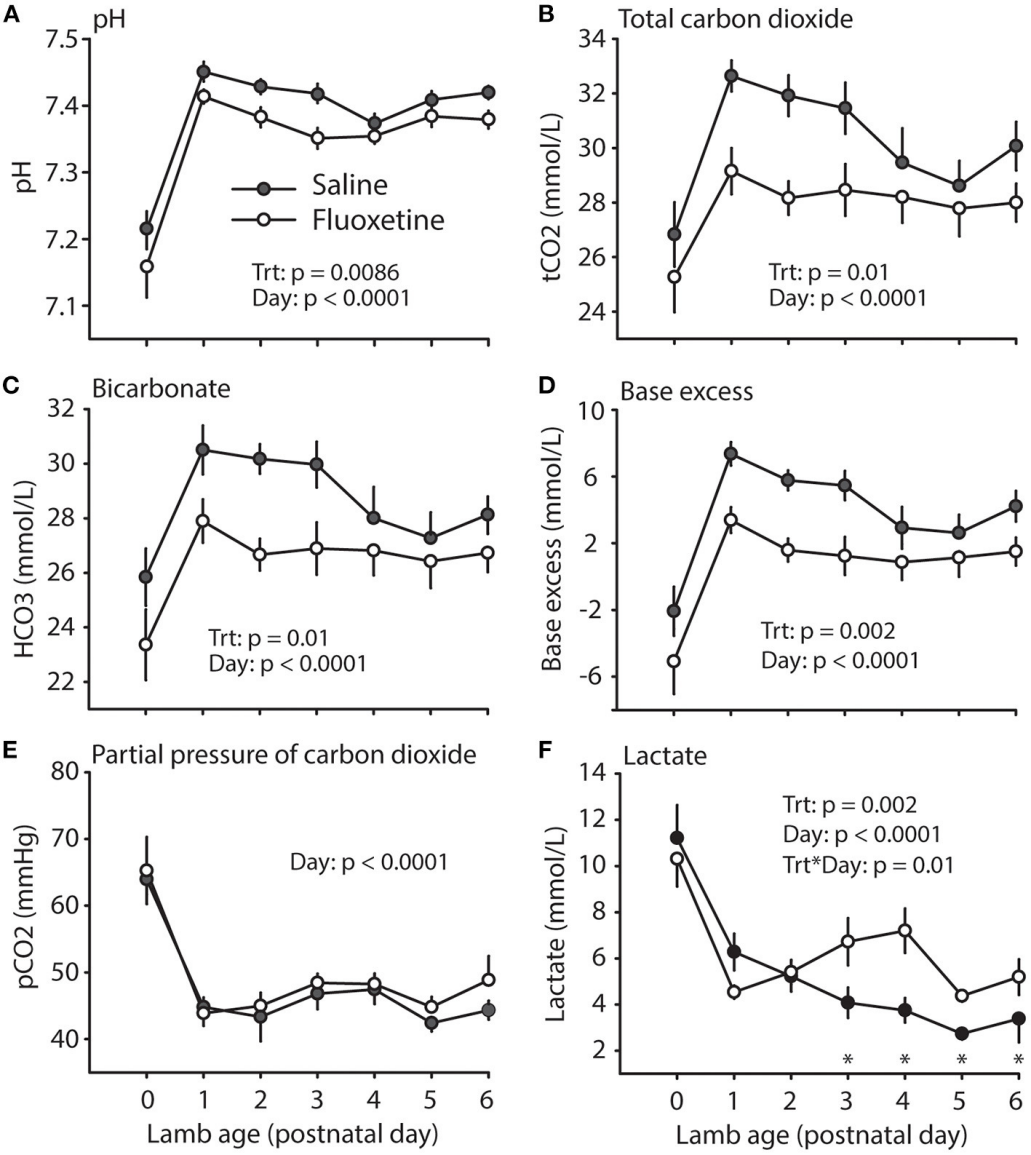
Lamb acid-base status. **(A)** pH. **(B)** Total carbon dioxide. **(C)** Bicarbonate. **(D)** Base excess. **(E)** Partial pressure of carbon dioxide. **(F)** Lactate. *Indicates significant difference between groups.

Fluoxetine treatment did not significantly affect maternal pH and blood gas status on postnatal days 0 (shortly after parturition) and 6 (Table 1). Maternal lactate was increased in the fluoxetine group only immediately after parturition. However, lambs born to fluoxetine-treated ewes had overall decreased blood pH during the evaluated period (Figure 5). Additionally, total carbon dioxide, bicarbonate and base excess were decreased in lambs from fluoxetine-treated ewes. For serum lactate, there were overall significant effects of treatment and day and an interaction indicating increased lactate in fluoxetine lambs on postnatal days 3–6.

**Table 1:** Maternal acid-base status.

**Outcomes**	**Saline** **(*n =* 10)**	**Fluoxetine** **(*n =* 9)**	***P*-value**
**Postnatal day 0**			
pH	7.508 ± 0.01	7.439 ± 0.04	0.14
Partial pressure of carbon dioxide, mmHg	33.1 ± 1.1	35.2 ± 3.7	0.6
Bicarbonate, mmol/L	23.5 ± 2.5	23.4 ± 1.6	0.9
Total carbon dioxide, mmol/L	27.2 ± 0.8	25.0 ± 1.3	0.2
Base excess, mmol/L	3.3 ± 0.9	0.0 ± 1.7	0.1
Lactate, mmol/L	3.2 ± 0.7	7.0 ± 1.6	0.02
**Postnatal day 6**			
pH	7.485 ± 0.01	7.478 ± 0.02	0.8
Partial pressure of carbon dioxide, mmHg	39.5 ± 1.2	39.3 ± 0.9	0.9
Bicarbonate, mmol/L	29.7 ± 0.7	29.0 ± 0.9	0.6
Total carbon dioxide, mmol/L	30.8 ± 0.8	30.1 ± 0.9	0.6
Base excess, mmol/L	6.2 ± 0.8	5.6 ± 1.2	0.9
Lactate, mmol/L	1.7 ± 0.4	2.8 ± 0.8	0.2

**Figure 6:**
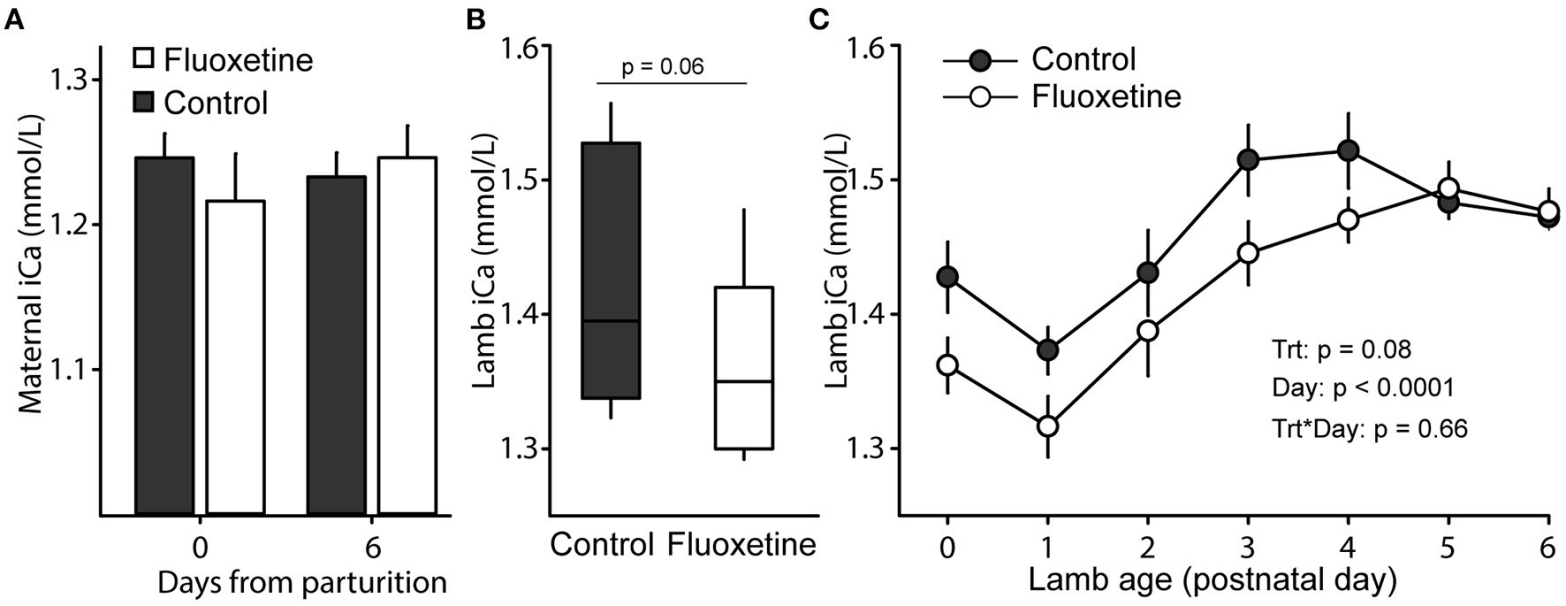
Ionized calcium (iCa) concentrations. **(A)** Maternal levels of iCa at parturition and 6 days postpartum. **(B)** Boxplot of neonatal concentrations of iCa after lambing. Boxplot depicts median, 10th, 25th, 75th, and 90th percentile. **(C)** Concentrations of iCa in lambs during days 0–6 of age.

Maternal concentrations of ionized calcium were not different between control and fluoxetine groups (Figure 6). Similarly, total calcium concentration was not different between groups in the colostrum (*P* > 0.1; 3.7 ± 0.4 vs. 2.5 ± 0.2 ng/dL) or milk on day 6 postpartum (*p* > 0.1; 1.8 ±. 0.2 vs. 1.5 ± 0.1 ng/dL). Nevertheless, ionized calcium in the newborn lamb was decreased (*P* = 0.06) at birth and overall (*P* = 0.08) during postnatal days 0–6 (Figure 6). An overall analysis from postnatal days 0–4 found ionized calcium concentrations were decreased (*P* = 0.01) in lambs born to fluoxetine-treated ewes than controls.

## Discussion

Understanding the effects of maternal medication on pregnancy complications and neonatal outcomes is vital to comprehensively assess the risk of perinatal exposure to psychotropic medication on maternal and newborn wellbeing during the peripartum period. Findings from the present study are especially relevant because they clearly establish a role for fluoxetine treatment during gestation on the occurrence of perinatal complications and extend previously known effects of *in utero* exposure to fluoxetine into the postpartum period. More specifically, findings from the present study support the hypotheses that fluoxetine treatment during late pregnancy (1) decreases gestation length, (2) causes IUGR (decreased placentome growth and decreased birthweight) and (3) affects neonatal health (neonatal acidemia, hyperlactemia, and hypocalcemia). Furthermore, the decreased capacity of the neonate to establish adequate acid-base balance in the present study extends previously reported short-term intrauterine fluoxetine-induced fetal acidemia (26) to neonatal acidemia during the first week of life in neonates exposed to fluoxetine *in utero*.

Increased plasma and/or placenta serotonin content are associated with placenta pathology and IUGR in humans and animal models: idiopathic IUGR in humans (10, 11, 27); serotonin or serotonin precursor treatment in rodents (28, 29); SSRI treatment in humans (4, 6, 15), mice (30–32), and sheep (present study); SERT null mouse model (33, 34). Increased serotonin signaling caused decreased blood perfusion to the placenta (13, 35) resulting in abnormal placenta function and growth (19). Similarly, women undergoing SSRI treatment during gestation (36) and pregnant rats treated with 5HTP (serotonin precursor) (28) exhibit decreased placental weights. In the present study, the functional area of the placenta was reduced due to fluoxetine treatment—a strong indication of IUGR (37, 38). The reduced uterine blood flow (26) and decreased placenta growth/size (28, 36) in addition to altered placenta morphology (15) caused by fluoxetine treatment might be the cause of placental insufficiency and IUGR resulting in decreased newborn weight. Placental insufficiency is an important cause of preterm birth (39). Accordingly, fluoxetine-induced placental insult leading to placental insufficiency is likely associated with the increased incidence of preterm birth in women and the shorter gestation length in sheep exposed to fluoxetine.

Fluoxetine-induced decrease in uterine artery blood flow has been associated with decreased partial pressure of oxygen and oxygen saturation of hemoglobin, as well as fetal acidemia accompanied by increased partial pressure of carbon dioxide (26). Although lambs are normally born in an acidotic state (40, 41), intrauterine exposure to fluoxetine caused further reductions in neonatal blood pH and this was maintained during the first week of life. Hypoxia-induced neonatal acidosis after parturition is a clinical indicator of placental insufficiency (42). Fluoxetine-induced fetal acidemia has similarities to respiratory acidosis since there is increased partial pressure of carbon dioxide perhaps due to reduced diffusion of carbon dioxide from fetal to maternal circulation related to reduced placental blood flow. Conversely, the postnatal acidemia is quite different since there is no change on partial pressure of carbon dioxide. In addition, the decreased bicarbonate and base excess are consistent with metabolic acidosis suggesting an effect of increased plasma serotonin on kidney function as it has been shown (28). Similarly, the increased lactate on postnatal days 3–6 may be related to altered renal function (28) or hypoxia due to respiratory distress commonly related to *in utero* exposure to SSRI (43).

The similar reduction in serum serotonin concentrations in ewes and lambs highlights the capacity of fluoxetine to inhibit fetal/neonatal SERT, thereby, increasing plasma serotonin and possibly giving rise to serotonin toxicity/syndrome in newborns exposed to serotonergic drugs as it has been reported (44–48). Other SSRI with reduced placental transfer may cause more mild symptoms (49). Additionally, although persistent pulmonary hypertension of the newborn is the most recognizable complication from *in utero* SSRI exposure (5, 18), we described less commonly reported SSRI-induced homeostatic imbalance—neonatal acidemia, hyperlactemia, and hypocalcemia. Neonatal providers should be aware of possible outcomes to discern between serotonin syndrome, drug withdraw, and other SSRI-related perinatal morbidity (45, 50).

Most studies in women have failed to determine whether adverse pregnancy outcomes are related to the use of SSRI or with depression itself (5). However, the observed outcomes in this ovine model clearly establishes a role for fluoxetine in perinatal complications independent of the effects of depression and highlights possible perinatal complications that arise from maternal use of fluoxetine. In a recent report, only moderate and high doses of SSRI taken during entire gestation were related to preterm birth while low doses or dose reduction/discontinuation during the first trimester did not (43). Along with the present results, it seems likely that SSRI exposure primarily during late pregnancy is the main driver of preterm birth. Of special relevance, <30 days of treatment during late gestation reduced pregnancy length and increased neonatal morbidity.

Although it was not investigated in the present study, SSRI use has been associated with other pregnancy complications such as increased risk of gestational hypertension, preeclampsia, and postpartum hemorrhage. Interestingly, SSRI-induced increased serotonin may be involved in the occurrence of these conditions. As a vasoactive hormone, increased plasma serotonin affect blood pressure and may lead to hypertension as reported in patients taking SSRI (51). Additionally, SSRI increases the risk for preeclampsia likely due to altering uteroplacental blood perfusion (52). Noteworthy, gestational hypertension, preeclampsia, and SSRI use during gestation increase the risk of preterm birth (2, 5, 51, 52). On a similar note, COVID-19 infection has also been associated with preeclampsia, preterm birth, and low birthweight in infants. It has been suggested that the effects of COVID-19 infection are likely associated with uteroplacental vasoconstriction and endothelial disfunction due to SARS-CoV-2 modulation of renin-angiotensin-aldosterone system by binding to angiotensin-converting enzyme 2 (53, 54). The pathophysiology of uteroplacental blood perfusion and its impact on preterm birth under different scenarios such as SSRI use, gestational hypertension, preeclampsia, and COVID-19 needs to be addressed to improve maternal and fetal health. Lastly, the decreased platelet serotonin content caused by SSRI use may be related to the increased risk for postpartum hemorrhage because platelet serotonin plays an important role in platelet aggregation and vasoconstriction (55, 56). Accordingly, SSRI use has been associated with abnormal bleeding (55).

The main strengths of this study are related to the animal model. Although rodent models are widely used in biomedical research, similarities between humans and sheep (number of fetuses per gestation, fetal intrauterine development, and stage of fetal organ maturation at birth) as opposed to humans and mice (57) make sheep a superb animal model for studies of pregnancy that may allow more direct translation to human medicine (14, 58, 59). Because this sheep model recapitulated several findings associated with fluoxetine exposure during pregnancy in women (3, 4, 6, 43), it should be considered for further investigation of the mechanistic effects of fluoxetine on pregnancy outcomes and neonatal heath. The ovine model has been widely used to study IUGR (14, 58, 59) but our study emphasizes the power of this model in understanding pharmaceutical effects on placental development, pregnancy, and neonatal outcomes. Specifically, our experiment further supports the use of the ovine model in translational pregnancy studies for investigating mechanistic actions of fluoxetine on the regulation of placental function and fetal development and to explore preclinical implementation of preventive therapies to overcome the adverse effects. Furthermore, the rapid onset (within 7 days) of reduction of placental size in this model can be useful to identify SSRI-dependent and independent early placental changes, physiologic mechanisms, and possibly placental markers of placental insufficiency that culminate with decreased placental growth, fetal growth restriction, and preterm birth for future clinical triage of pregnancies at greater risk.

A limitation of our study is the difference in normal blood values (pH, lactate, blood gases, calcium) between human babies and lambs which challenges a direct comparison between species. Additionally, we did not investigate the underlying cause of fluoxetine-induced fetal/neonatal acidemia and hypocalcemia. Lastly, while the intravenous administration of the drug allowed consistent delivery of the desired amount of drug it does not represent the pharmacokinetics of absorption of fluoxetine in humans taking the drug orally.

In conclusion, maternal fluoxetine treatment during late gestation reduced placental growth, caused IUGR, and decreased gestation length similar to the effects of fluoxetine treatment in pregnant, depressed women. Additionally, lambs exposed to fluoxetine *in utero* exhibited metabolic acidemia and hypocalcemia during the first week of life, as observed in preterm and IUGR babies. However, more than recapitulating findings in women, we established a role for fluoxetine in perinatal complications and neonatal morbidity in our non-depressed sheep model shedding light on the interpretation of the effects of SSRI on pregnancy outcomes in women previously obscured by the effects of depression itself.

## Data availability statement

The original contributions presented in the study are included in the article, further inquiries can be directed to the corresponding author.

## Ethics statement

All protocols were approved by the University of Wisconsin-Madison Institutional Animal Care and Use Committee under the Protocol Number A006302-A02.

## Author contributions

RD: conceptualization, methodology, validation, formal analysis, investigation, data curation, writing—original draft, writing—review and editing, visualization, and project administration. AB: validation, investigation, data curation, writing—review and editing, and project administration. MC: validation, investigation, data curation, and writing—review and editing. MW and LH: conceptualization, methodology, resources, writing—review and editing, visualization, supervision, and funding acquisition. All authors contributed to the article and approved the submitted version.

## Funding

Funding was provided by R01HD0947450-03W1 to LH.

## Conflict of interest

The authors declare that the research was conducted in the absence of any commercial or financial relationships that could be construed as a potential conflict of interest.

## Publisher's note

All claims expressed in this article are solely those of the authors and do not necessarily represent those of their affiliated organizations, or those of the publisher, the editors and the reviewers. Any product that may be evaluated in this article, or claim that may be made by its manufacturer, is not guaranteed or endorsed by the publisher.

## References

[1] Diav-Citrin O Ornoy A. Selective serotonin reuptake inhibitors in human pregnancy: to treat or not to treat? Obstet Gynecol Int. (2012) 2012:1–12. 10.1155/2012/698947 22190957 PMC3236415

[2] Oberlander TF Warburton W Misri S Aghajanian J Hertzman C. Neonatal outcomes after prenatal exposure to selective serotonin reuptake inhibitor antidepressants and maternal depression using population-based linked health data. Arch Gen Psychiatry. (2006) 63:898–906. 10.1001/archpsyc.63.8.898 16894066

[3] Tran H Robb AS. SSRI use during pregnancy. Semin Perinatol. (2015) 39:545–7. 10.1053/j.semperi.2015.08.010 26428019

[4] Velasquez JC Goeden N Bonnin A. Placental serotonin: implications for the developmental effects of SSRIs and maternal depression. Front Cell Neurosci. (2013) 7:1–7. 10.3389/fncel.2013.00047 23630464 PMC3632750

[5] Alwan S Friedman JM Chambers C. Safety of selective serotonin reuptake inhibitors in pregnancy: a review of current evidence. CNS Drugs. (2016) 30:499–515. 10.1007/s40263-016-0338-3 27138915

[6] Zhao XF Liu Q Cao SX Pang JY Zhang HJ Feng TT. A meta-analysis of selective serotonin reuptake inhibitors (SSRIs) use during prenatal depression and risk of low birth weight and small for gestational age. J Affect Disord. (2018) 241:563–70. 10.1016/j.jad.2018.08.061 30153640

[7] Mohammad-Zadeh LF Moses L Gwaltney-Brant SM. Serotonin: a review. J Vet Pharmacol Ther. (2008) 31:187–99. 10.1111/j.1365-2885.2008.00944.x 18471139

[8] Blardi P De Lalla A Leo A Auteri A Iapichino S Di Muro A. Serotonin and fluoxetine levels in plasma and platelets after fluoxetine treatment in depressive patients. J Clin Psychopharmacol. (2002) 22:131–6. 10.1097/00004714-200204000-00005 11910257

[9] Ranzil S Walker DW Borg AJ Wallace EM Ebeling PR Murthi P. The relationship between the placental serotonin pathway and fetal growth restriction. Biochimie. (2019) 161:80–7. 10.1016/j.biochi.2018.12.016 30605696

[10] Ranzil S Ellery S Walker DW Vaillancourt C Alfaidy N Bonnin A. Disrupted placental serotonin synthetic pathway and increased placental serotonin: Potential implications in the pathogenesis of human fetal growth restriction. Placenta. (2019) 84:74–83. 10.1016/j.placenta.2019.05.012 31176514 PMC6724713

[11] Hernandez-Rodriguez J Meneses L Herrera R Manjarrez G. Another abnormal trait in the serotonin metabolism path in intrauterine growth-restricted infants. Neonatology. (2009) 95:125–31. 10.1159/000153096 18776726

[12] Lang U Prada J Clark KE. Systemic and uterine vascular response to serotonin in third trimester pregnant ewes. Eur J Obstet Gynecol Reprod Biol. (1993) 51:131–8. 10.1016/0028-2243(93)90025-8 8119459

[13] Honey DP Robson JM Sullivan FM. Mechanism of inhibitory action of 5-hydroxytryptamine on placental function. Am J Obstet Gynecol. (1967) 99:250–7. 10.1016/0002-9378(67)90328-6 6039068

[14] Morrison JL. Sheep models of intrauterine growth restriction: fetal adaptations and consequences. Clin Exp Pharmacol Physiol. (2008) 35:730–43. 10.1111/j.1440-1681.2008.04975.x 18498533

[15] Levy M Kovo M Miremberg H Anchel N Herman HG Bar J. Maternal use of selective serotonin reuptake inhibitors (SSRI) during pregnancy-neonatal outcomes in correlation with placental histopathology. J Perinatol. (2020) 40:1017–24. 10.1038/s41372-020-0598-0 31988450

[16] Park S-Y Kim MY Kim YJ Chun YK Kim HS Kim HS. Placental pathology in intrauterine growth retardation. Korian J Pathol. (2002) 36:30–7.17883125

[17] Marcorelles P. Placental features in intrauterine growth retardation. Journal De Gynecologie Obstetrique Et Biologie De La Reproduction. (2013) 42:996–1007. 10.1016/j.jgyn.2013.09.021 24210718

[18] Norby U Forsberg L Wide K Sjors G Winbladh B Kallen K. Neonatal morbidity after maternal use of antidepressant drugs during pregnancy. Pediatrics. (2016) 138:e20160181. 10.1542/peds.2016-0181 27940758

[19] Rosenfeld CS. Placental serotonin signaling, pregnancy outcomes, and regulation of fetal brain development. Biol Reprod. (2020) 102:532–8. 10.1093/biolre/ioz204 31711155 PMC7443348

[20] Bonnin A Goeden N Chen K Wilson ML King J Shih JC. A transient placental source of serotonin for the fetal forebrain. Nature. (2011) 472:347–52. 10.1038/nature09972 21512572 PMC3084180

[21] Pacher P Kecskemeti V. Cardiovascular side effects of new antidepressants and antipsychotics: new drugs, old concerns? Curr Pharm Design. (2004) 10:2463–75. 10.2174/1381612043383872 15320756 PMC2493295

[22] Ghavamabadi RT Taghipour Z Hassanipour M Khademi M Shariati M. Effect of maternal fluoxetine exposure on lung, heart, and kidney development in rat neonates. Iran J Basic Med Sci. (2018) 21:417–21. 10.22038/IJBMS.2018.27203.6650 29796227 PMC5960760

[23] NRC. Nutrient Requirements for Ruminants: Sheep, Goats, Cervids and New World Camelids. Washington, DC: National Academic Press (2007).

[24] Stankiewicz T Blaszczyk B Udala J Chundekkad P. Morphometric measurements of the umbilical cord and placentomes and Doppler parameters of the umbilical artery through ultrasonographic analysis in pregnant sheep. Small Ruminant Res. (2020) 184:106043. 10.1016/j.smallrumres.2019.106043

[25] Connelly MK Weaver SR Kuehnl JM Fricke HP Klister M Hernandez L. Elevated serotonin coordinates mammary metabolism in dairy cows. Physiol Rep. (2021) 9:e14798. 10.14814/phy2.14798 33835711 PMC8034258

[26] Morrison JL Chien C Riggs KW Gruber N Rurak D. Effect of maternal fluoxetine administration on uterine blood flow, fetal blood gas status, and growth. Pediatr Res. (2002) 51:433–42. 10.1203/00006450-200204000-00007 11919327

[27] Hernandez J Manjarrez G Chagoya G. Newborn humans and rats malnourished in utero: free plasmal-tryptophan, neutral amino acids and brain serotonin synthesis. Brain Res. (1989) 488:1–13. 10.1016/0006-8993(89)90687-2 2743105

[28] Salas SP Giacaman A Romero W Downey P Aranda E Mezzano D. Pregnant rats treated with a serotonin precursor have reduced fetal weight and lower plasma volume and kallikrein levels. Hypertension. (2007) 50:773–9. 10.1161/HYPERTENSIONAHA.107.094540 17646571

[29] Van Cauteren H Vandenberghe J Marsboom R. Protective activity of ketanserin against serotonin-induced embryotoxicity and teratogenicity in rats. Drug Dev Res. (1986) 8:179–85. 10.1002/ddr.430080121

[30] Bauer S Monk C Ansorge M Gyamfi C Myers M. Impact of antenatal selective serotonin reuptake inhibitor exposure on pregnancy outcomes in mice. Am J Obstet Gynecol. (2010) 203:375.e1–4. 10.1016/j.ajog.2010.05.008 20541736

[31] Ali M Deen S-E El Menshawy O Bakry S. Fluoxetine Hcl induced intrauterine foetal growth retardation and skeletal malformation in pregnant mice. Egypt J Hospital Med. (2002) 6:63–79. 10.21608/ejhm.2002.18857

[32] Domingues RR Fricke HP Sheftel CM Bell AM Sartori LC Manuel RS. Effect of low and high doses of two selective serotonin reuptake inhibitors on pregnancy outcomes and neonatal mortality. Toxics. (2022) 10:11. 10.3390/toxics10010011 35051053 PMC8780128

[33] Hadden C Fahmi T Cooper A Savenka AV Lupashin VV Roberts DJ. Serotonin transporter protects the placental cells against apoptosis in caspase 3-independent pathway. J Cell Physiol. (2017) 232:3520–9. 10.1002/jcp.25812 28109119 PMC5522371

[34] Domingues RR Wiltbank MC Hernandez LL. Pregnancy complications and neonatal mortality in a serotonin transporter null mouse model: insight into the use of selective serotonin reuptake inhibitor during pregnancy. Front Med. (2022) 2022:626. 10.3389/fmed.2022.848581 35360732 PMC8960382

[35] Robson JM Sullivan FM. Mechanism of lethal action of 5-hydroxytryptamine on the foetus. J Endocrinol. (1963) 25:553. 10.1677/joe.0.0250553 13974493

[36] Kivisto J Lehto SM Halonen K Georgiadis L Heinonen S. Maternal use of selective serotonin reuptake inhibitors and lengthening of the umbilical cord: indirect evidence of increased foetal activity-a retrospective cohort study. PLoS ONE. (2016) 11:e0154628. 10.1371/journal.pone.0154628 27128030 PMC4851376

[37] Constancia M Hemberger M Hughes J Dean W Ferguson-Smith A Fundele R. Placental-specific IGF-II is a major modulator of placental and fetal growth. Nature. (2002) 417:945–8. 10.1038/nature00819 12087403

[38] Salavati N Smies M Ganzevoort W Charles AK Erwich JJ Plosch T. The possible role of placental morphometry in the detection of fetal growth restriction. Front Physiol. (2019) 9:1884. 10.3389/fphys.2018.01884 30670983 PMC6331677

[39] Morgan TK. Role of the placenta in preterm birth: a review. Am J Perinatol. (2016) 33:258–66. 10.1055/s-0035-1570379 26731184

[40] Vannucchi CI Rodrigues JA Silva LCG Lucio CF Veiga GAL. A clinical and hemogasometric survey of neonatal lambs. Small Ruminant Res. (2012) 108:107–12. 10.1016/j.smallrumres.2012.05.013

[41] Arfuso F Giannetto C Giudice E Assenza A Piccione G. Daily dynamic changes of blood acid-base status and vital parameters in lambs and goat kids over the first seven days after birth. Small Ruminant Res. (2021) 197:106340. 10.1016/j.smallrumres.2021.106340

[42] MacDonald TM Hui L Tong S Robinson AJ Dane KM Middleton AL. Reduced growth velocity across the third trimester is associated with placental insufficiency in fetuses born at a normal birthweight: a prospective cohort study. Bmc Medicine. (2017) 15:164. 10.1186/s12916-017-0928-z 28854913 PMC5577811

[43] Bandoli G Chambers CD Wells A Palmsten K. Prenatal antidepressant use and risk of adverse neonatal outcomes. Pediatrics. (2020) 146. 10.1542/peds.2019-2493 32513841 PMC7329255

[44] Morris R Matthes J. Serotonin syndrome in a breast-fed neonate. Case Rep. (2015) 2015:bcr2015209418. 10.1136/bcr-2015-209418 25948853 PMC4434373

[45] Brajcich MR Palau MA Messer RD Murphy ME Marks J. Why the maternal medication list matters: neonatal toxicity from combined serotonergic exposures. Pediatrics. (2021) 147:e20192250. 10.1542/peds.2019-2250 33504611

[46] Isbister GK Dawson A Whyte IM Prior FH Clancy C Smith AJ. Neonatal paroxetine withdrawal syndrome or actually serotonin syndrome? Arch Dis Child Fetal Neonatal Ed. (2001) 85:F147–8. 10.1136/fn.85.2.F145g 11561552 PMC1721292

[47] Haddad PM Pal BR Clarke P Wieck A Sridhiran S. Neonatal symptoms following maternal paroxetine treatment: Serotonin toxicity or paroxetine discontinuation syndrome? J Psychopharmacol. (2005) 19:554–7. 10.1177/0269881105056554 16166193

[48] Eleftheriou G Butera R Cottini FC Bonati M Farina M. Neonatal toxicity following maternal citalopram treatment. Fetal Pediatr Pathol. (2013) 32:362–6. 10.3109/15513815.2013.768743 23438790

[49] DeVane CL. Metabolism and pharmacokinetics of selective serotonin reuptake inhibitors. Cell Mol Neurobiol. (1999) 19:443–66. 10.1023/A:1006934807375 10379420 PMC11545448

[50] Laine K Heikkinen T Ekblad U Kero P. Effects of exposure to selective serotonin reuptake inhibitors during pregnancy on serotonergic symptoms in newborns and cord blood monoamine and prolactin concentrations. Arch Gen Psychiatry. (2003) 60:720–6. 10.1001/archpsyc.60.7.720 12860776

[51] Watts SW Morrison SF Davis RP Barman SM. Serotonin and blood pressure regulation. Pharmacol Rev. (2012) 64:359–88. 10.1124/pr.111.004697 22407614 PMC3310484

[52] Guan HB Wei Y Wang LL Qiao C Liu CX. Prenatal selective serotonin reuptake inhibitor use and associated risk for gestational hypertension and preeclampsia: a meta-analysis of cohort studies. J Womens Health. (2018) 27:791–800. 10.1089/jwh.2017.6642 29489446

[53] Wei SQ Bilodeau-Bertrand M Liu S Auger N. The impact of COVID-19 on pregnancy outcomes: a systematic review and meta-analysis. Cmaj. (2021) 193:E540–E8. 10.1503/cmaj.202604 33741725 PMC8084555

[54] Wang M Zhang B Jin L. Female fertility under impact of COVID-19 pandemic: a narrative review. Exp Rev Molecu Med. (2021) 23:1–21. 10.1017/erm.2021.19

[55] Meijer WE Heerdink ER Nolen WA Herings RM Leufkens HG Egberts AC. Association of risk of abnormal bleeding with degree of serotonin reuptake inhibition by antidepressants. Arch Intern Med. (2004) 164:2367–70. 10.1001/archinte.164.21.2367 15557417

[56] Hanley GE Smolina K Mintzes B Oberlander TF Morgan SG. Postpartum hemorrhage and use of serotonin reuptake inhibitor antidepressants in pregnancy. Obstet Gynecol. (2016) 127:553–61. 10.1097/AOG.0000000000001200 26855096

[57] Hemberger M Hanna CW Dean W. Mechanisms of early placental development in mouse and humans. Nat Rev Genet. (2020) 21:27–43. 10.1038/s41576-019-0169-4 31534202

[58] Beede KA Limesand SW Petersen JL Yates DT. Real supermodels wear wool: summarizing the impact of the pregnant sheep as an animal model for adaptive fetal programming. Anim Front. (2019) 9:34–43. 10.1093/af/vfz018 31608163 PMC6777506

[59] Swanson AM David AL. Animal models of fetal growth restriction: Considerations for translational medicine. Placenta. (2015) 36:623–30. 10.1016/j.placenta.2015.03.003 25819810

